# Epicardial adipose tissue in type 1 diabetes mellitus: a systematic review and meta-analysis

**DOI:** 10.1186/s43044-025-00666-8

**Published:** 2025-07-14

**Authors:** Basavaraj G. Sooragonda, Vanshika Karnwal, Vikneswaran Gunaseelan, Ameya Joshi, Kaushik Biswas, Mohan T. Shenoy, Rakesh Anbazhagan

**Affiliations:** 1 Narayana Health Bengaluru, Bengaluru, India; 2 Narayana Hrudayalaya Institute of Allied Health Sciences, Bengaluru, India; 3 Bhaktivedanta Hospital And Research Institute, Mira-Bhayandar, India; 4 Medica Superspecialty Hospital, Kolkata, India; 5 Sree Gokulam Medical College and Research Foundation, Thiruvananthapuram, India; 6 coGuide Academy, Bengaluru, India

**Keywords:** Epicardial adipose tissue, Type 1 diabetes mellitus, Cardiovascular diseases, Systematic review, Meta-analysis

## Abstract

**Background:**

Epicardial adipose tissue (EAT) is a metabolically active visceral fat depot surrounding the myocardium and evidence suggests its potential role in the development of cardiovascular complications in type 1 diabetes mellitus (T1DM). This systematic review and meta-analysis aimed to: (1) quantify EAT measurements (thickness and volume) in patients with T1DM, and (2) compare EAT measurements between T1DM patients and healthy controls.

**Methods:**

A comprehensive literature review was conducted using a systematic search strategy to identify studies that measured EAT thickness or volume in T1DM patients in PubMed, Embase, Cochrane Library, Web of Science, and CINAHL. Studies were included if they: (1) involved patients with T1DM, (2) reported EAT measurements using imaging techniques, and (3) were published in English. Case reports and reviews were excluded. Two independent reviewers performed study selection, data extraction, and quality assessment. Study quality was evaluated using the Newcastle–Ottawa Scale (NOS). Statistical heterogeneity was assessed using I^2^ statistics. Meta-analysis was performed using a random effect model.

**Results:**

A total of nine studies involving 285 and 233 participants measuring thickness and volume with T1DM were included. The pooled mean EAT thickness was 5.81 mm (95%CI: 4.30, 7.32 mm), and the pooled mean EAT volume was 56.84cm^3^ (95%CI: 34.05, 79.63cm^3^). Significant heterogeneity was observed between the volume and thickness of EAT among people with T1DM (I^2^ = 99% for volume and 95% for thickness). Subgroup analysis revealed a mean difference of 2.12 mm (95%CI: 0.82, 3.43 mm) in EAT thickness between T1DM and control groups.

**Conclusions:**

Our findings indicate increased EAT measurements in T1DM patients compared to healthy individuals, suggesting EAT’s potential involvement in T1DM-related cardiovascular issues.

## Background

Epicardial adipose tissue (EAT) is a visceral fat depot distributed along the coronary arteries, Found between the myocardium and the visceral layer of the pericardium [1].

Once considered a passive structural component, EAT is now recognized as a metabolically dynamic organ that secretes various bioactive molecules, including pro-inflammatory adipokines and cytokines [2, 3]. The anatomical proximity of EAT to the myocardium and the lack of fascia separating them allows for the free exchange of these secreted factors, potentially influencing cardiac structure and function [4]. Research has linked increased EAT volume to several cardiovascular issues, such as coronary artery disease, irregular heart rhythms, and cardiac failure [1, 5].

While the role of EAT in the pathogenesis of type 2 diabetes mellitus (T2DM) and its cardiovascular complications has been extensively studied. [6, 7], the literature examining the role of EAT in T1DM is notably limited. [8] Further, these studies have shown inconsistent results, varying methodologies, and limited longitudinal data. This knowledge gap is particularly concerning, given that T1DM patients face a two–fourfold higher risk of cardiovascular diseases compared to the general population, though the exact mechanisms remain incompletely understood. [9] The paucity of comprehensive research on EAT in T1DM represents a significant gap in our understanding of cardiovascular risk factors in this population.

The emerging evidence suggests that chronic low-grade inflammation and systemic insulin resistance may contribute to the accelerated development of cardiovascular complications in T1DM [9, 10]. Given the pro-inflammatory and metabolic properties of EAT, it is plausible that increased EAT volume could play a role in the pathogenesis of T1DM and its associated cardiovascular risks [11].

The reviews have also shown that EAT accumulation in T1DM likely represents a maladaptive response rather than an adaptive one [12]. In the normal physiological state, EAT serves as a local energy source and provides mechanical protection for the heart [13]. However, in T1DM, the chronic hyperglycemic environment and insulin deficiency appear to trigger pathological changes in EAT, leading to dysfunction in its secretory profile [14]. This maladaptive response is characterized by increased pro-inflammatory adipokine secretion and reduced anti-inflammatory factors, potentially contributing to local inflammation and accelerated atherosclerosis [15, 16]. Understanding this maladaptive nature of EAT accumulation in T1DM is crucial for developing targeted therapeutic strategies.

This paper aims to synthesize the existing evidence on the relationship between EAT and T1DM. By pooling data from individual studies, we aim to provide a comprehensive assessment of the magnitude and direction of this association.

## Methods

### Study design and registration

We conducted this systematic review and meta-analysis following PRISMA (Preferred Reporting Items for Systematic Reviews and Meta-Analyses) guidelines to assess the relationship between EAT and T1DM using the. Our review protocol was pre-registered with PROSPERO, the International Prospective Register of Systematic Reviews (Registration Number CRD42024534550).

### Eligibility criteria

For this systematic review, we have included observational studies (cross-sectional, case control, or cohort designs) examining patients with clinically diagnosed Type 1 Diabetes Mellitus (T1DM) across all age groups. Primary outcome measures comprised quantitative assessments of epicardial adipose tissue (EAT) thickness (mm) or volume (cm^3^) obtained through standardized imaging modalities (echocardiography or computed tomography). Studies were excluded if they incorporated mixed diabetes populations without discrete T1DM data stratification, consisted of case reports, reviews, or conference abstracts, lacked quantitative EAT measurements, or contained overlapping patient populations with other included studies. 

### Information sources and Search strategy

We conducted a systematic literature review utilizing multiple electronic databases, including PubMed, Embase, Cochrane Library, Web of Science, and CINAHL. Our search encompassed all relevant publications up to February 2024, with language restrictions applied to include only English-language articles. To ensure a thorough and inclusive search strategy, we employed a range of techniques including the use of synonyms, keyword variations, truncation symbols, wildcard characters, and proximity operators.

The search strategy was structured around two primary concepts: epicardial adipose tissue and type 1 diabetes mellitus. For each database, we utilized appropriate subject headings (such as MeSH terms in PubMed and Emtree in Embase) alongside free-text terms to capture relevant studies.

In PubMed, we combined MeSH terms like “Epicardial Adipose Tissue” with title/abstract searches for related terms such as “Epicardial Fat” and “Pericoronary Adipose Tissue.” Similarly, for type 1 diabetes, we used the MeSH term “Diabetes Mellitus, Type 1” along with various synonyms in the title/abstract fields. This approach was adapted for each database to align with its specific indexing system and search capabilities.

The individual concept searches were then combined using Boolean operators to identify studies at the intersection of epicardial adipose tissue and type 1 diabetes. The initial search yielded 12 results in PubMed, 2 in Cochrane Library, 45 in Embase, 12 in Web of Science, and 39 in CINAHL.

To enhance the comprehensiveness of our review, we conducted a manual screening of the bibliographies of selected articles, aiming to identify any pertinent studies potentially overlooked in the primary database searches. Furthermore, we supplemented our systematic search with a targeted exploration using Google Scholar to capture any gray literature or recently published articles not yet indexed in the major databases.

### Selection process

Following our comprehensive database search, we employed a systematic approach to manage and screen the identified literature. The search outputs from all databases were consolidated into a dedicated Zotero library. We then utilized Zotero’s automatic duplicate identification feature to remove initial duplicates, merging records as necessary.

The resulting list was exported to Rayyan software for further refinement. In Rayyan, we conducted an additional round of duplicate removal using its automatic detection function, followed by a quick manual review of bibliographic details to ensure no duplicates were overlooked.

With a final de-duplicated list established, we proceeded to Level 1 screening. This involved two independent researchers carefully examining the titles and abstracts of each entry, identifying studies that potentially met our inclusion criteria. In cases where the two reviewers disagreed, a third reviewer was consulted to resolve the discrepancy.

Studies that passed Level 1 screening were then subjected to Level 2 screening. We retrieved full-text articles for all shortlisted studies. Two reviewers, working independently and blinded to each other’s decisions, thoroughly read these full-text articles. Their assessments were recorded directly in Rayyan. As with Level 1, any conflicts in decision-making were resolved through discussion, with input from a third reviewer when necessary.

**Fig. 1:**
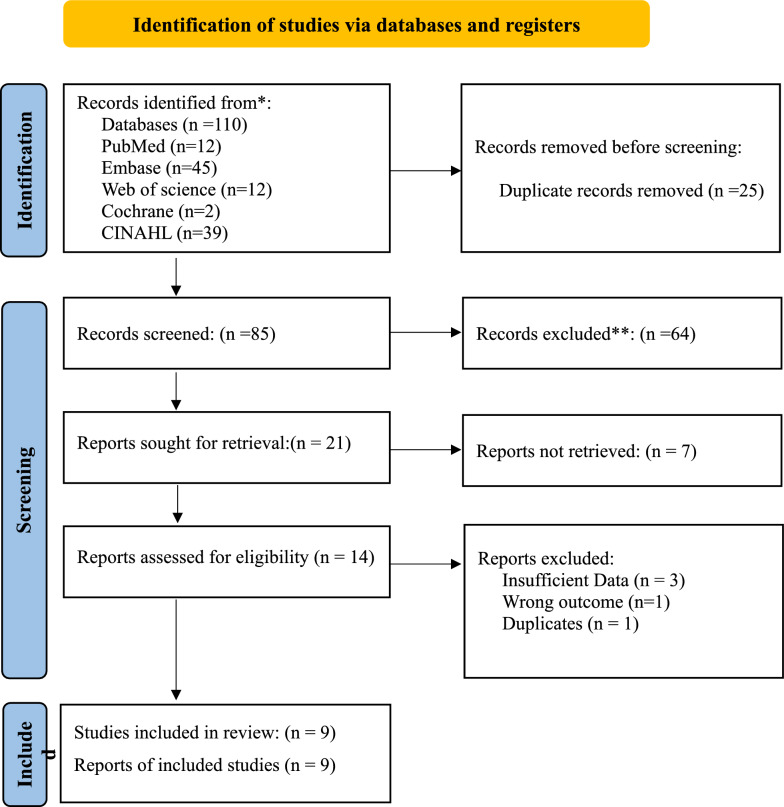
PRISMA flowchart. *Consider, if feasible to do so, reporting the number of records identified from each database or register searched (rather than the total number across all databases/registers). **If automation tools were used, indicate how many records were excluded by a human and how many were excluded by automation tools

The PRISMA flowchart (Fig. 1) provides a clear visual representation of our study selection journey.

### Data collection process

We conducted a meticulous data extraction process to assess the relationship between epicardial adipose tissue (EAT) volume and type 1 diabetes mellitus (T1DM). Two independent reviewers (BGS, and VK) used a standard data extraction form to ensure comprehensive and consistent data collection. We endeavored to obtain missing data by contacting study authors directly. A double review process was implemented to minimize errors and ensure data completeness. This approach enhanced the reliability and validity of our extracted data, providing a robust foundation for subsequent analysis of the EAT volume-T1DM risk relationship.

### Data items

Studies with the following primary outcome measures were included in the review—Author name, year, region, population, sample size, Age, Duration of T1DM, Imaging modality, EAT/EFT thickness and EAT/EFT volume. Inter-reviewer discrepancies were resolved through collaborative consensus among all authors.

### Quality appraisal

The included studies were critically appraised using Newcastle–Ottawa Scale (NOS) to evaluate the methodological. Two independent reviewers (BS and VK) assessed each study across three domains: selection, comparability, and outcome. The NOS evaluation considered crucial factors including sample representativeness, exposure ascertainment methodology, and outcome assessment protocols [17].

### Data analysis

Descriptive statistics were utilized for data synthesis, with frequency statistics applied to comparable quantitative data across studies. Statistical analyses was done using RStudio Desktop Version 2023.03.0 + 386 (RStudio Team, 2023). To assess statistical heterogeneity, we employed the Cochrane-Q test and I^2^ statistic. The selection between fixed effect and random effect models was contingent upon heterogeneity levels: a fixed effect model was applied when *P* > 0.05 in the Cochrane-Q test or I^2^ < 50%, while a random effect model was used when *P* < 0.05 or I^2^ > 50%. For categorical variables, we calculated pooled proportions with 95% confidence intervals. This methodological approach ensured a comprehensive and statistically sound synthesis of the available evidence, accounting for between-study variability and providing a robust foundation for our findings.

## Results

### Description of studies

The search strategy yielded 110 studies. However, on removing the duplicates, the number was reduced to 85 studies. On screening, 64 studies were considered as irrelevant and 21 were assessed for level-2 screening. Finally, nine studies were included in our meta-analysis studies that measured the thickness or volume of EAT among people with T1DM [1, 18–25]

The key features of the included studies are summarized in Table 1. The sample sizes ranged from 30 to 148 participants across the studies. Six studies [1, 18, 21–23, 25] evaluated EAT (EAT) thickness using echocardiographic measurements, while three studies [19, 20, 24] assessed EAT (EAT) volume through computed tomography (CT) imaging in a total of 285 and 233 participants, respectively. The study population consisted of individuals with T1DM.

**Table 1:** Characteristic of included studies

Author	Country	N	Measurement tool	Group	Age	BMI Kg/m^2^	EFT/ EAT	Overall quality
Ahmad et al. [2022]	Egypt	100	Echocardiography (Echo)	T1DM	12.90 ± 1.30	20.57 ± 1.7	6.60 ± 0.71	Good Quality
				Control	12.96 ± 1.12	20.33 ± 0.69	3.83 ± 0.35	
Svanteson et al. [2019]	Norway	148	Coronary computed tomography angiography (CT)	T1DM	61.3 ± 7.1	25.8 ± 3.9	52.3(36.1–65.5)	Good Quality
				Control	62.3 ± 6.8	25.5 ± 4.2	55 (38.3–79.6) cm3, Median (IQR)	
de Gonzalo-Calvo et al. [2018]	Spain	73	Computed tomography(CT)		47.1 ± 8.6	27.0 ± 4.6	80.2 ± 49.0Cm3	Fair Quality
Keles et al. [2016]	Turkey	80	Transthoracic echocardiography (Echo)	T1DM	34 (29–39)	24.1(22.3–26.7)	0.7 (0.6–0.9)	Good Quality
				Control	32(28–37.5)	26.7(21.8–30.2)	0.6 (0.5–0.7)	
Aslan et al. [2015] [17]	Turkey	112	Echocardiography (Echo)	T1DM	30.6 ± 10.3	23.31 ± 2.73	3.56 ± 0.48	Good Quality
				Control	32.4 ± 8.5	24.19 ± 2.70	3.03 ± 0.48	
Iacobellis et al. [2014] [22]	USA	30	Transthoracic echocardiography (Echo)	T1DM	52.8 ± 12	27.8 ± 5.2	7.2 ± 2.1	Good Quality
				Control	53 ± 9	27.4 ± 4.1	4.9 ± 2.5	
Yazıcı et al. [2011] [18]	Turkey	79	Transthoracic echocardiography (Echo)	T1DM	30.8 ± 7.7	24.7 ± 3.8	3.30 ± 1.06	Good Quality
				Control	29.9 ± 4.9	24.6 ± 3.0	2.30 ± 0.34 mm	
Colom et al. [2018] [19]	Spain	72	Computed tomography (CT)		47.1 ± 8.7	27.0 ± 4.7/27.9	40.47 ± 22.18 cm3/m2	Fair Quality
ElBaky et al. [2023] [20]	Egypt	97	Echocardiography (Echo)	T1DM	12.82 ± 3.24	21.15 ± 4.10	7.01 ± 1.85	Good Quality
				Control	11.65 ± 3.54	18.72 ± 4.10	2.16 ± 0.58	

### EAT thickness and volume

**Fig. 2:**
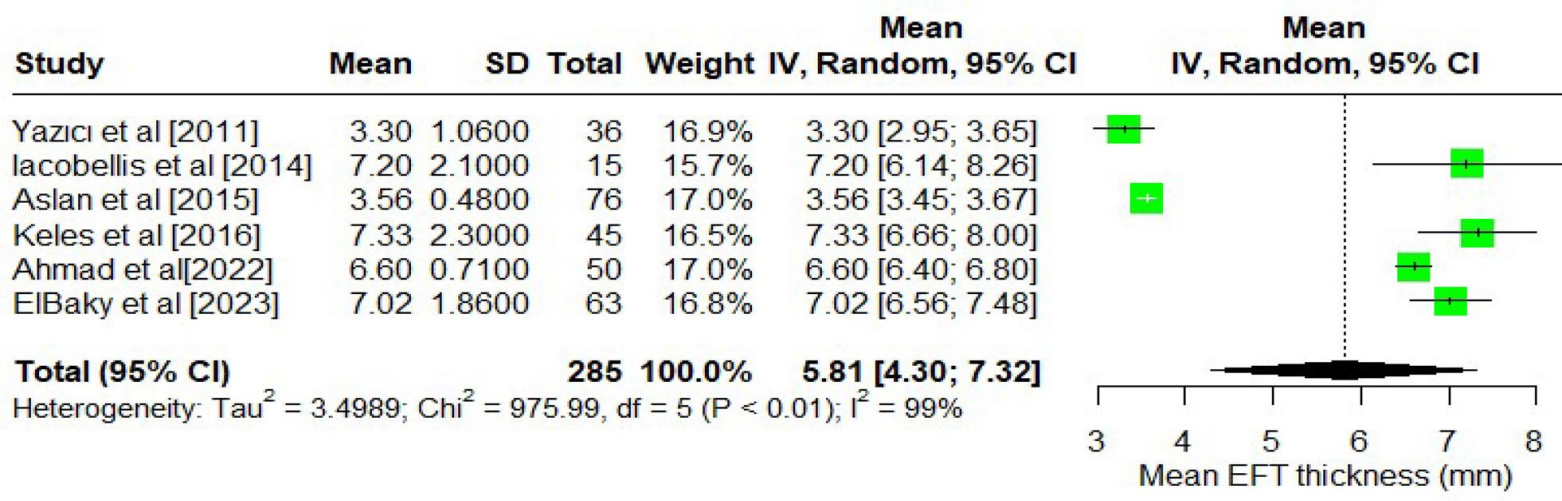
Mean EAT thickness among T1DM patients

**Fig. 3:**
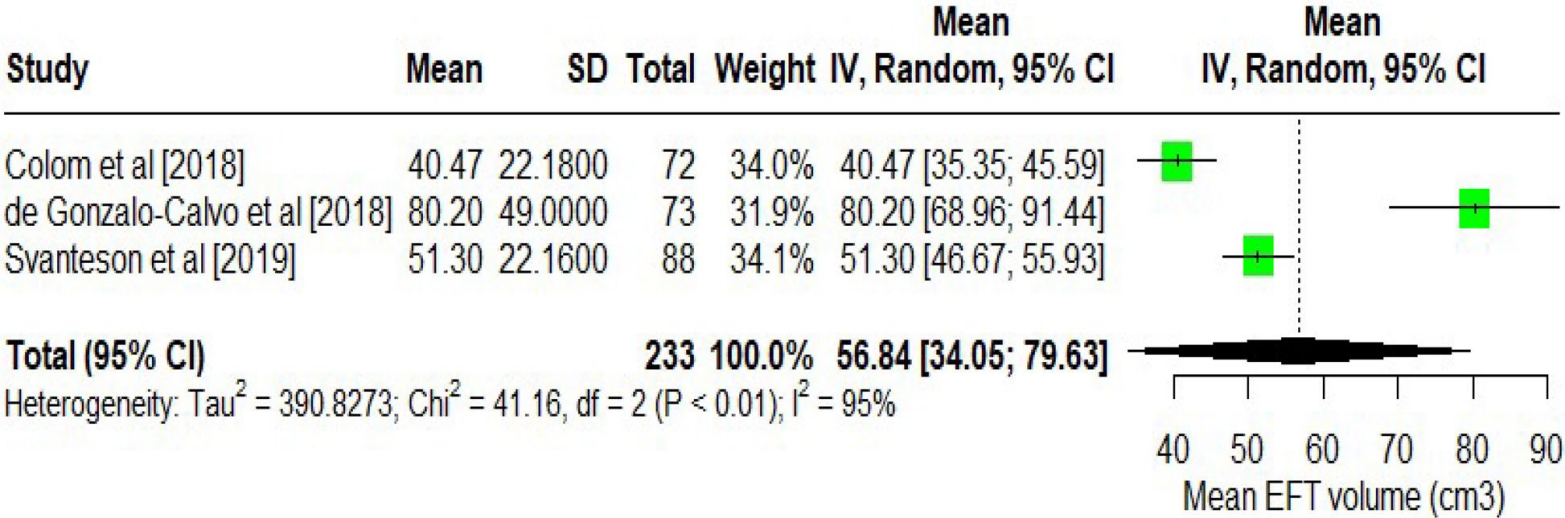
Mean EAT volume between T1DM patients

The pooled mean EAT (Fig. 2) thickness across the included studies was 5.81 mm (95% CI: 4.30, 7.32 mm). The analysis was done by pooling the thickness measured using echocardiograms. Significant heterogeneity existed between the study (I^2^ = 99% and *p*-value < 0.001). The pooled mean of epicardial tissue volume (Fig. 3) was 56.84 cm^3^ (95% CI: 34.05, 79.63 cm^3^). Similarly, for the epicardial tissue volume analysis, significant heterogeneity was present (*p* < 0.01; I^2^ = 95%). Due to the substantial heterogeneity observed in both analyses, a random effect was employed to pool the thickness of EAT and volume measurements across studies.

### Comparative analysis of T1 DM and control group

**Fig. 4:**
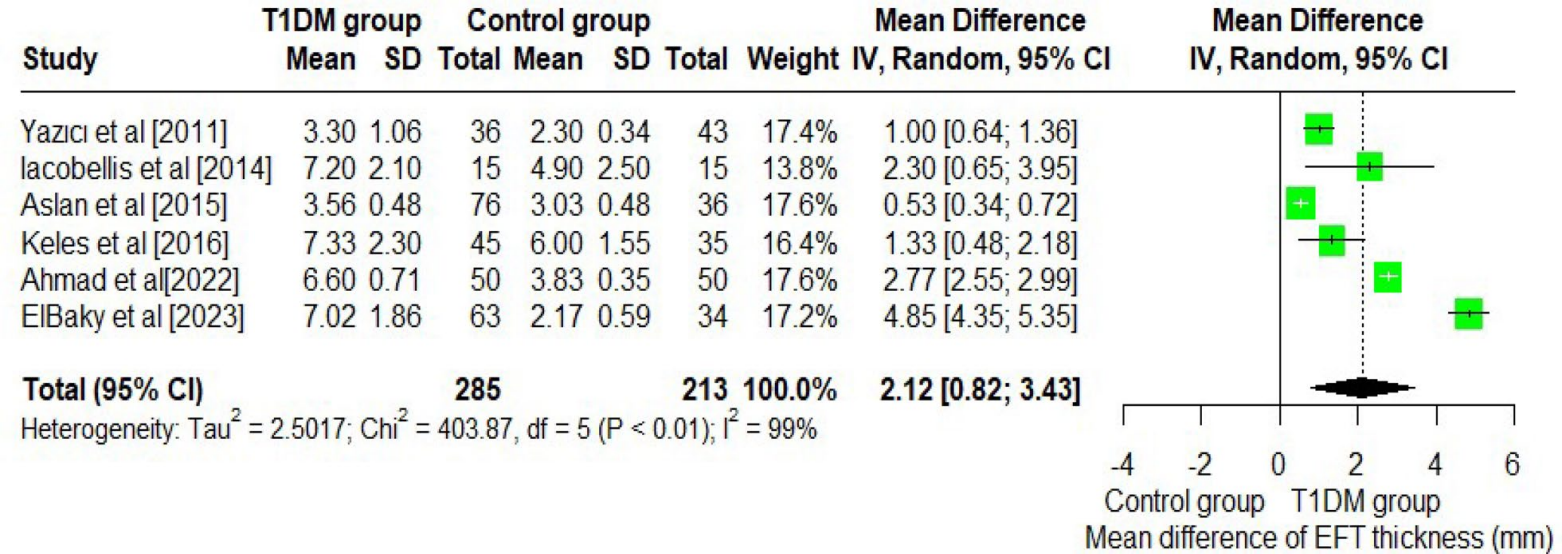
Comparison of EAT thickness among T1DM vs healthy controls

The subgroup analysis was done based on the EAT thickness among type 1 diabetes mellitus vs healthy (control) group. The pooled mean difference in EAT thickness between the T1DM and healthy (control) group, calculated using a random effect model, was 2.12 mm (95% CI: 0.82, 3.43 mm). This suggests that individuals with T1DM had significantly higher thickness compared to healthy controls, with a mean difference of approximately 2.12 mm. (Fig. 4).

Considering the higher heterogeneity (I^2^ = 98.8% and *p*-value < 0.001), a random effect model was employed to substantiate it.

While this meta-analysis found increased EAT (EAT) in T1DM patients compared to controls, there was a notable lack of studies specifically measuring EAT volume in T1DM patients. The majority of included studies assessed EAT thickness by echocardiography rather than volumetric measurements.

## Discussion

Type 1 diabetes mellitus (T1DM) is an autoimmune condition characterized by insulin deficiency when beta cells in the pancreas stop producing insulin [26]. EAT has been a subject of interest in context with T1DM.

## Summary of main findings

This systematic review and meta-analysis focused on the influence of EAT in T1DM. Our analysis included nine studies that measured the thickness and volume of among individuals with T1DM. It concluded that T1DM patients have higher EAT thickness and volume compared to healthy controls, with a pooled mean EAT of 5.81 mm (95% CI: 4.30, 7.32 mm) and a pooled mean volume of 56.84 cm^3^ (95% CI: 34.05, 79.63 cm^3^). Subgroup analysis revealed a significant difference in EAT between T1DM and control groups, with a mean difference of 2.12 mm (95% CI: 0.82, 3.43 mm).

### Interpretation and comparison with previous studies

The findings of our systematic review were consistent with previous studies that have reported increased EAT in individuals with T1DM. Several studies, including Keles et al.(2016) [21], Iacobellis et al.(2014) [27], and Yazici et al.(2011) [23], have also observed significantly higher EAT in T1DM patients compared to controls. The increase in EAT in T1DM has been attributed to insulin resistance, which is a common feature of the disease. Insulin resistance is known to promote the accumulation of visceral and ectopic fat, including EAT, which may contribute to the development of cardiovascular complications in T1DM.

Studies that reported increased EAT in T1DM:

Chambers et al. (2019) reported that T1DM cases exhibited significantly higher EAT compared to controls [28], and a similar study done by Guney et al. (2020) has also reported significantly higher EAT in both adult and pediatric T1DM populations compared to controls [29].

### Heterogeneity in findings

A significant finding in our meta-analysis was the substantial heterogeneity observed across studies (I^2^ = 99% for thickness and 95% for volume measurements). Several factors likely contribute to this heterogeneity:

1. *Patient characteristics*: The included studies featured populations with varying demographic profiles. The mean age of participants ranged from 12.9 years to 61.3 years, representing both pediatric and adult populations with fundamentally different metabolic profiles. This wide age range likely contributes to variations in EAT measurements, as EAT accumulation typically increases with age [30, 31].
2. *Disease duration and severity*: Studies included patients with varying durations of T1DM, ranging from recently diagnosed cases to those with decades of disease progression. The cumulative effect of glycemic variability and metabolic derangements over time likely influences EAT deposition differently across these populations [32].
3. *Regional and ethnic variations*: The included studies were conducted across diverse geographical regions including Egypt, Norway, Spain, Turkey, and the USA. Ethnic and regional differences in body fat distribution and cardiometabolic risk profiles, if well-documented may explain some variations in EAT measurements [33].
4. *Study design heterogeneity*: The included studies employed different methodological approaches, ranging from cross-sectional to case control designs, with varying inclusion and exclusion criteria that may have selected for different patient phenotypes.

### Impact of confounding variables

Our analysis revealed several important confounding variables that likely influence the relationship between T1DM and EAT:

1. *Body Mass Index (BMI)*: The mean BMI across studies ranged from 20.57 kg/m^2^ to 27.8 kg/m^2^, representing populations from normal weight to overweight. Given that BMI is independently associated with EAT deposition, this variation likely influenced the reported measurements [34] .

### Influence of imaging modalities on results

The included studies employed two different imaging techniques to assess EAT: echocardiography (six studies) and computed tomography (three studies). This methodological difference likely contributed substantially to the observed heterogeneity:

1. *Operator-dependent variability*: Echocardiographic measurements are notably operator-dependent, with potential variations in the precise location and angle of measurements. This technical variability may have contributed to differences in reported EAT thickness values.
2. *Standardization challenges*: The lack of universally standardized protocols for EAT measurement across both imaging modalities presents a significant challenge for meta-analyses in this field. Future research would benefit from consensus guidelines on EAT assessment techniques.[35].

### Strengths and limitations

A notable strength of our study is the systematic and comprehensive approach employed in identifying and synthesizing the available evidence on role of EAT in T1DM. We adhered to the PRISMA guidelines, ensuring the methodological rigor and transparency of our review process. Furthermore, our meta-analysis included relatively large sample size, enhancing the statistical power and precision of our estimates. Additionally, risk of bias assessment using the Newcastle–Ottawa scale allowed us to evaluate the quality of the included studies and account for potential biases in our analysis.

However, several limitations should be acknowledged. Even though the majority of the included studies were of good quality based on the Newcastle–Ottawa scale assessment, some studies were rated as having a fair quality, indicating potential biases that could have influenced their results. The included studies were observational and the potential for residual confounding cannot be ruled out. The assessment of EAT was performed using different imaging modalities (echocardiography and computed tomography), which may introduce variability in the measurements.

### Clinical implications

The findings of our systematic review have important clinical implications for the management of individuals with T1DM. The increased EAT observed in this population may contribute to the accelerated development of cardiovascular complications, highlighting the need for early risk stratification and preventive strategies. Measuring EAT by noninvasive imaging techniques like echocardiography or computed tomography could potentially serve as a valuable tool for identifying T1DM patients at higher cardiovascular risk and guiding personalized treatment approaches.

## Conclusion and recommendation

In conclusion, our systematic review provides evidence of significantly increased epicardial adipose tissue in T1DM patients compared to healthy controls, suggesting its potential role as a cardiovascular risk marker in this population. The anatomical proximity of EAT to the myocardium and its pro-inflammatory properties may contribute to accelerated cardiovascular pathology in T1DM. However, before incorporating EAT measurement into routine clinical practice, several challenges must be addressed, including standardization of measurement protocols, establishment of reference values, validation of its incremental predictive value beyond traditional risk factors, and cost-effectiveness analyses. Currently, EAT quantification may be most appropriate in research settings or as a complementary assessment in selected high-risk T1DM patients. Future prospective studies are essential to determine whether interventions targeting EAT reduction translate to improved cardiovascular outcomes and whether this promising biomarker merits integration into routine cardiovascular risk assessment in T1DM patients.

## Data Availability

No datasets were generated or analysed during the current study.

## Abbreviations

EAT: Epicardial adipose tissue
T1DM: Type 1 diabetes mellitus
T2DM: Type 2 diabetes mellitus
NOS: Newcastle–Ottawa Scale
PRISMA: Preferred reporting items for systematic reviews and meta-analyses
EFT: Epicardial fat tissue
CT: Computed tomography
BMI: Body mass index
